# Enhanced intracellular delivery via coordinated acoustically driven shear mechanoporation and electrophoretic insertion

**DOI:** 10.1038/s41598-018-22042-0

**Published:** 2018-02-27

**Authors:** J. Mark Meacham, Kiran Durvasula, F. Levent Degertekin, Andrei G. Fedorov

**Affiliations:** 1 Department of Mechanical Engineering and Materials Science, Washington University in St. Louis, St. Louis, MO 63130 USA; 2 OpenCell Technologies, Inc, St. Louis, MO 63108 USA; 3 School of Electrical and Computer Engineering, Georgia Institute of Technology, Atlanta, GA 30332 USA; 4 Parker H. Petit Institute for Bioengineering and Bioscience, Georgia Institute of Technology, Atlanta, GA 30332 USA; 5 G. W. Woodruff School of Mechanical Engineering, Georgia Institute of Technology, Atlanta, GA 30332 USA

## Abstract

Delivery of large and structurally complex target molecules into cells is vital to the emerging areas of cellular modification and molecular therapy. Inadequacy of prevailing *in vivo* (viral) and *in vitro* (liposomal) gene transfer methods for delivery of proteins and a growing diversity of synthetic nanomaterials has encouraged development of alternative physical approaches. Efficacy of injury/diffusion-based delivery via shear mechanoporation is largely insensitive to cell type and target molecule; however, enhanced flexibility is typically accompanied by reduced gene transfer effectiveness. We detail a method to improve transfection efficiency through coordinated mechanical disruption of the cell membrane and electrophoretic insertion of DNA to the cell interior. An array of micromachined nozzles focuses ultrasonic pressure waves, creating a high-shear environment that promotes transient pore formation in membranes of transmitted cells. Acoustic Shear Poration (ASP) allows passive cytoplasmic delivery of small to large nongene macromolecules into established and primary cells at greater than 75% efficiency. Addition of an electrophoretic action enables active transport of target DNA molecules to substantially augment transfection efficiency of passive mechanoporation/diffusive delivery without affecting viability. This two-stage poration/insertion method preserves the compelling flexibility of shear-based delivery, yet substantially enhances capabilities for active transport and transfection of plasmid DNA.

## Introduction

The cell membrane is a selectively permeable barrier between a cell and its environment, regulating passage of material into and out of the cell. Membrane transport is fundamental to the intrinsic functioning of the cell with several natural mechanisms (e.g., passive diffusion, active and co-transport, and endocytosis/exocytosis) permitting cellular uptake and secretion of small and large molecules^1^. Macromolecular delivery is also critical to the advancement of biomedical science, playing a key role in basic research, diagnostic and therapeutic applications and industrial bioproduction^2,3^. Historically, significant effort has focused on strategies for effective DNA and RNA delivery; however, the predominant methods for *in vivo* (viral) and *in vitro* (liposomal) transfection are not well-suited to delivery of proteins, small molecules, quantum dots and other nanoparticles of interest in emerging clinical and laboratory applications (e.g., cell reprogramming^4–6^, genome editing^7^ and intracellular labeling^8^).

Many small lipophilic molecules spontaneously cross biological membranes. This is not true of larger macromolecules, which require alternative means to enter the cell interior. Ideal delivery systems also protect materials from cytoplasmic degradation, convey materials to a target location, and facilitate action on that target^9–12^. The advantages and limitations of viral and non-viral chemical vectors are well documented^2,3,13–20^. Of note, the effectiveness of chemical methods is significantly diminished in difficult-to-transfect primary cells (stem cells and immune cells)^2,3^. Physical (non-viral, non-chemical) approaches to delivery include direct insertion and field-mediated disruption of the cell membrane (electrical, mechanical/acoustic, shear, optical or thermal). Microinjection bypasses various biological barriers to delivery providing direct access to the cytoplasm or nucleus regardless of cell type or target molecule^21,22^. In practice, this unique capability is negated by the low throughput of the method. Field-mediated membrane poration has supplanted chemical methods in many delivery applications, particularly those involving nongene target molecules and primary cells. Electroporation is most widely accepted with demonstrated efficacy of DNA^23,24^, RNA^25,26^ and even protein delivery^27^; however, this method can produce unacceptable levels of cell death, DNA damage and electric field-induced agglomeration of certain nanomaterials^8^. While electroporation and sonoporation are relatively mature technologies, the last decade has witnessed the emergence of several alternative injury/diffusion-based delivery methods including optoporation^28^, thermoporation^29^, high-frequency acoustic transfection^30^, hypersonic poration^31^, and continuous-flow, shear-based mechanoporation^32–35^. These technologies are often amenable to miniaturization, enabling rapid advancement of intracellular delivery applications through introduction of microfluidics and nanotechnology^2,3^.

Shear-based methods induce transient pore formation in the cell membrane through exposure to mechanical stresses in confined flow geometries. Hallow *et al*.^32^ observed small molecule uptake after forcing suspended cells and target molecules through three-dimensional (3D) constrictions under regulated pressure-driven flow. In a parallel effort, Zarnitsyn *et al*.^33^ emphasized the importance of precise control over both shear stress magnitude and exposure duration on cell uptake of biomolecules. Converging nozzle-like channels were used to achieve DNA transfection via focused acoustic pressure driven cell mechanoporation. More recently, Sharei *et al*.^34–36^ have demonstrated the insensitivity of these methods to cell type and target molecule, providing additional evidence to support their potential as a universal route to *in vitro* and *ex vivo* delivery. Efficiency of these methods is comparable to microinjection due to single-cell scale treatment; however, parallel arrays of flow constrictions in microchannels (2D) or orifice plates (3D) yield much higher throughput. This facile parallelization and scale up are crucial to therapeutic applications and cell-based biomanufacturing, where sample sizes can exceed billions of cells^2^. Delivery of small molecules, proteins, siRNA, and quantum dots into primary and stem cells at up to 1 × 10^5^ cells/s has been demonstrated^32–34^.

Delivery of macromolecules such as nucleic acids to primary cells *ex vivo* is a critical component of many new cell-based therapies such as adoptive T-cell immunotherapy. For example, chimeric antigen receptor (CAR)-modified T cells have been targeted to CD19 to successfully treat patients with relapsed or refractory B-cell acute lymphoblastic leukemia (B-ALL)^37^. There is a major potential for extension of CAR-T cell therapy to other hematologic malignancies (e.g., multiple myeloma) and many solid tumors; however, existing approved CAR-T cell therapies and those under development all use effective yet undesirable viral vectors for nucleic acid delivery. Direct delivery of nucleic acids as described in this work offers a compelling alternative that avoids the inherent shortcomings of viral vectors.

The shear mechanoporation method first reported by Zarnitsyn *et al*.^33^ achieved ~85% delivery of the small molecule calcein via diffusive uptake alone. Although the level of calcein delivery was remarkable, inadequate delivery of larger molecules (no nongene macromolecules; ~2% transfection efficiency for plasmid DNA) was a significant limitation. In 2014, Meacham *et al*.^2,38^ introduced a method to enhance intracellular delivery by combining shear mechanoporation with electric field induced molecular uptake. In this approach, high shear mechanical stimuli reversibly porate cells as they are forced through microscopic orifices by an acoustically-driven pressure field. Mechanically porated cells and charged target molecules are then exposed to an electric field that is below the injury threshold. Shear-based poration followed by electrophoresis of DNA into the cell interior is found to significantly increase transfection efficiency over mechanoporation alone (from 13–57% to 28–87%), greatly expanding the applicability of this method (as was recently further substantiated by Ding *et al*.^39^). Like other physical methods, temporary pores can permit passive diffusion of target materials into the cytosol. Orifice diameter roughly delineates upper and lower bounds on shear rate and treatment time, and flow speed (as a function of acoustic drive amplitude) allows fine tuning of treatment parameters. This unique actuation mechanism provides access to a larger parameter space than other shear-based techniques^32,34–36,39^. The array format is easily scaled up or down, and pyramidal tapered nozzles minimize opportunities for clogging, which can lead to failure of 2D microchannel-based approaches^34–36,39^. As a permeabilization/diffusion-based method, our primary advantage is an ability to efficiently deliver large macromolecules (e.g., 2 MDa FITC-dextran at >75% efficiency) into the cytoplasm of established and primary cells, which benefits applications utilizing protein, small molecules and other nanomaterials. DNA transfection is inherently more challenging than nongene delivery. Here we discuss the mechanism of this combined mode operation in detail, including in-depth characterization of capabilities for delivery of plasmid DNA.

## Results

### Macromolecule Delivery and Gene Transfer Technique

**Figure 1:**
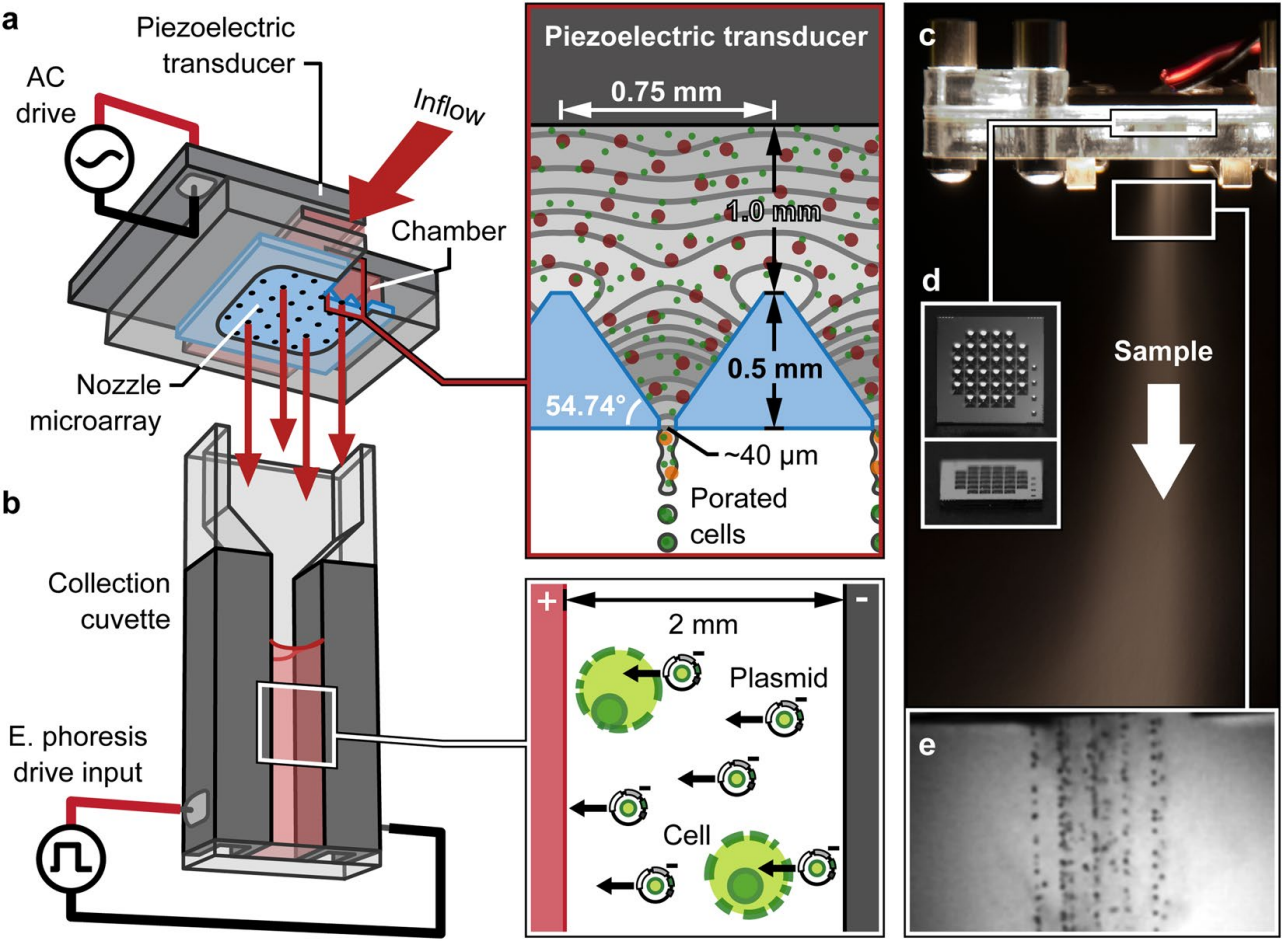
Combined mechanoporation/electrophoresis gene transfer method and system operation: **(a)** schematic of assembled Acoustic Shear Poration (ASP) module illustrating resonant acoustic field focusing that drives sample ejection and cell mechanoporation, **(b)** electrophoresis collection cuvette for active insertion of plasmid DNA, **(c)** sample misting at a drive frequency of 1.25 MHz, **(d)** 32-nozzle silicon ASP microarray, and **(e)** high-speed imaging of jet breakup during ejection from an array of 42 μm orifices at 1.13 MHz.

Figure 1 illustrates coupling of the Acoustic Shear Poration (ASP) technology to an electrophoresis collection cuvette for active insertion of charged molecules (e.g., negatively charged DNA) into cells. ASP comprises a piezoelectric transducer for acoustic wave generation, a chamber containing sample mixture, and a planar array of acoustic horn structures for focused application of mechanical stimuli. When driven at particular resonant frequencies of the fluid-filled horn structures, focused acoustic waves establish a favorable pressure gradient at the nozzle apices driving fluid transport through cell-sized orifices (Fig. 1, Supplementary Text and Supplementary Figs S1 and S2). As a consequence, suspended cells are exposed to mechanical forces that induce temporary and reversible poration of the cell membrane^33^. The chamber geometry and speed of sound characteristic of the fluid sample dictate operating frequencies, with typical chamber geometries yielding ejection in the 0.5 to 2.0 MHz range^33,40,41^. Under these conditions (orifice diameter *d* > 10 μm, *f* = 0.5–2.0 MHz), interactions of inertial, capillary and viscous effects dictate that fluid exits the orifices as a continuous jet (with subsequent downstream breakup, see Fig. 1e)^42^. It is important to note that the acoustic field itself is not sufficient to disrupt the cell membrane; membrane poration (and uptake) are observed only after exposure to the high-shear environment of the nozzle orifice.

Silicon microarrays contain 32 pyramidal nozzles with ~750-μm side-length square bases tapering to 20–50-μm diameter orifices (Fig. 1d). The orifice size of a given array is 2–3× the treated cell diameter. Each array is embedded in an acrylic/polycarbonate cartridge that includes a variable volume (100–800 μL, ~50 μL dead volume) sample reservoir. Piezoelectric actuation naturally empties a prescribed volume at a rate of 50–100 μL/s (50,000–400,000 cells/s at typical concentrations of 1–4 × 10^6^ cells/mL) (Supplementary Text and Supplementary Fig. S2). For passive macromolecule delivery by diffusion, sample is collected directly into a 1.5-mL microcentrifuge tube. Active insertion by electrophoresis is achieved using a standard 2-mm gap width electroporation cuvette and custom signal waveform.

### Underlying Poration Mechanism

**Figure 2:**
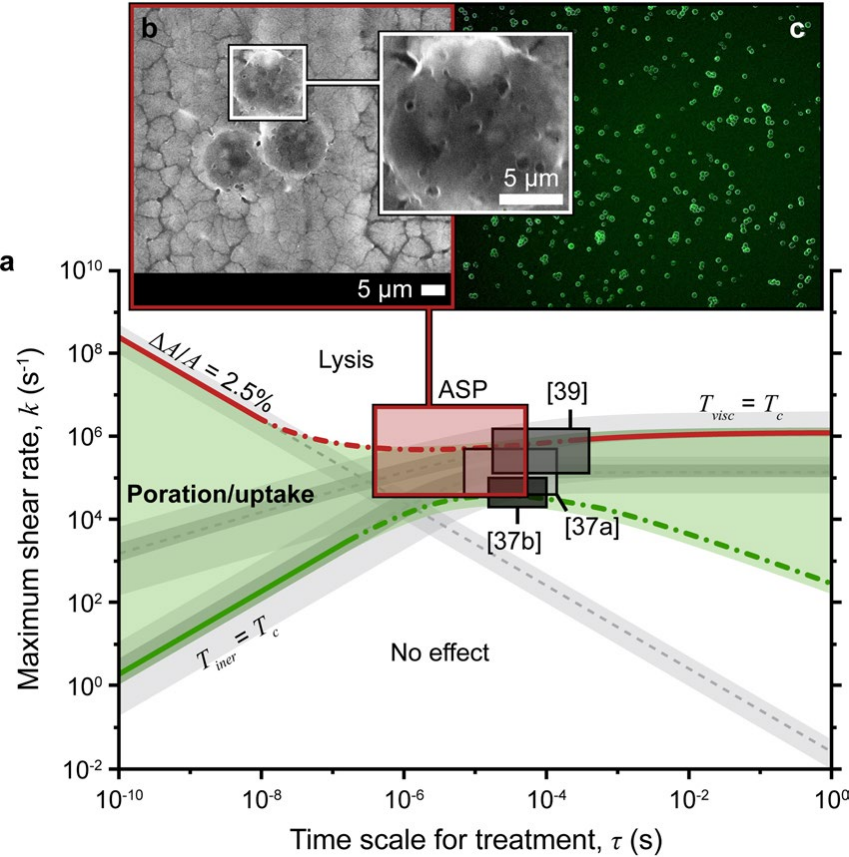
Expected ASP cell treatment outcomes: **(a)** regime map including domains of no effect, poration and lysis for HEK 293 A and Jurkat cells (lower and upper limits of the gray bands correspond to 293 A and Jurkat cells, respectively). Predicted attainable treatment domains are highlighted for ASP and three other shear-based mechanoporation methods ([37a] cylindrical orifice plate; [37b] tapered orifice array; and [39] microchannel constriction)^32,34^. **(b)** environmental scanning electron micrograph (ESEM) of hydrated porated 293 A cells (scale bars are 5 μm), and **(c)** representative calcein uptake in 293 A cells after ASP treatment (*d* = 30 μm, *f* = 1.25 MHz).

Exposure time *τ* and shear rate *k* predict cell treatment outcomes after ASP processing^33,43^. The relative magnitudes of these parameters delineate domains of no effect, reversible or irreversible poration, lysis and death for a particular cell type. A regime map valid for human embryonic kidney (HEK 293 A, cell radius *r*_*c*_ = 8 μm) and Jurkat (*r*_*c*_ = 6 μm) cells is shown in Fig. 2a [domain boundaries adapted from Lokhandwalla and Sturtevant^43^]. During passage of the cell through the orifice, fluid particle inertia and viscous stresses deform the cell membrane placing it under tension that acts to balance one or both of these effects. Experimental investigation of various biomembrane compositions indicates that rupture tension threshold lies between 1 and 25 mN/m^44–47^. In the low-*τ* region where inertia dominates, this corresponds to a critical areal strain Δ*A*/*A* of 1–5%. Under the viscous mode of deformation (high-*τ* region), lysis is expected if the membrane tension due to tangential viscous stresses *T*_*visc*_ exceeds the critical tension for rupture *T*_*c*_. In the absence of data specific to the cells used in the present study, Fig. 2a assumes values of 2.5% for the critical areal strain and 10 mN/m for the critical tension.

As undeformed cells are accelerated from the sample chamber toward the nozzle orifice, the resultant shear deforms cells causing tension buildup in their membranes. Onset of membrane permeabilization occurs if this tension *T*_*iner*_ exceeds the critical tension *T*_*c*_ defined above [solution of the equation *ρr*_*c*_^3^(*k*/*τ* + *k*^2^) = 0.01^33,43^; upper and lower limits of the gray bands in Fig. 2a correspond to Jurkat and HEK 293 A cells, respectively]. As referenced above, the transition from reversible to irreversible poration and cell lysis is represented by the lines labelled Δ*A*/*A* = 2.5% [solution of *kτ* = 0.025] and *T*_*visc*_ = *T*_*c*_ [solution of *ρr*_*c*_^2^(*ν*/*τ*)^1/2^ + *ρνkr*_*c*_ = 0.01^33,43^] for the inertial and viscous modes of deformation, respectively. Domain boundaries of the regime map depend on the cell type and liquid medium, i.e., radius *r*_*c*_, critical tension *T*_*c*_, and critical areal strain Δ*A*/*A* are properties of the cells; and *ρ* and *ν* are the density and kinematic viscosity of the liquid (treated as water, *ρ* = 1000 kg/m^3^ and *ν* = 1 × 10^−6^ m^2^/s).

Attainable hydrodynamic field parameters *k* and *τ* are specific to a particular mechanoporation method. For the shear mechanoporation method described herein, the velocity *U* and orifice diameter *d* are used to determine the time scale for treatment (*τ* ~ *d*/*U*) and maximum shear rate (*k* ~ *U*/(*d*/2))^33^. The resultant *k*-*τ* space for the ASP system is highlighted in Fig. 2a (d = 20–50 μm, *U* = 1–50 m/s). For the ASP device of Fig. 1, diameter *d* is the characteristic length used to determine both *k* and *τ*; however, the entire *k*-*τ* space can be accessed by uncoupling the field parameters through fabrication of a short channel at the nozzle tips (i.e., to adjust treatment time without affecting shear rate)^33^. Although approximate, scale analysis provides a physically accurate prediction of expected treatment outcomes ranging from poration and uptake to lysis and cell death (Fig. 2b,c). Orifice size dictates the spatial extent of the effective shear field, and an orifice diameter 2–3× that of the treated cell type allows access to the largest area within the poration/uptake domain (e.g., ASP30 30-μm orifice for 12 μm Jurkat and 16 μm HEK 293 A cells; ASP20 20-μm orifice for 9 μm peripheral blood mononuclear cells, PBMC). For a given ASP microarray, the characteristic jet velocity increases with increasing piezoelectric voltage amplitude at a fixed frequency of operation providing fine adjustment of treatment parameters.

Treatment domains for other shear-based mechanoporation methods (calculated in a similar manner; Supplementary Text) are also provided in Fig. 2 for reference^32,34^. The ASP ability to indirectly control jet velocity via piezoelectric drive voltage enables an order of magnitude higher maximum shear rate than that of systems driven by compressed gas or syringe pump. Further, to achieve adequate shear rates, some methods rely on narrow, sub cell-sized constrictions, which lowers throughput and may encourage clogging. Expanded ASP capability (i.e., the accessibility of higher shear operation) may be important for treatment of smaller cell types and/or cells with larger characteristic critical areal strain and/or membrane tension.

### Intracellular Delivery by Passive Diffusion

The mechanism by which shear-based methods effect membrane disruption differs markedly from that of electroporation. Electro-permeabilization occurs when the transmembrane potential induced by an external field exceeds a threshold value. The extent of poration is restricted to the poles of the cell, and only pores at the pole facing the cathode are large enough to accept macromolecules^2,48–50^. Conversely, though the flow field is not expected to impose uniform shear on the cell periphery, our results suggest that shear-based mechanoporation promotes more evenly distributed membrane fenestra. To assess differences between ASP- and electroporation-mediated uptake by passive diffusion, we investigated delivery of various cargo molecules (623 Da calcein, and 70 kDa, 500 kDa and 2 MDa fluorescein isothiocyanate (FITC)-labelled dextran) into two established cell types (HEK 293 A and Jurkat). A qualitative comparison was conducted using fluorescence microscopy (Supplementary Text and Supplementary Fig. S3), and delivery efficiency was quantified by flow cytometry.

**Figure 3:**
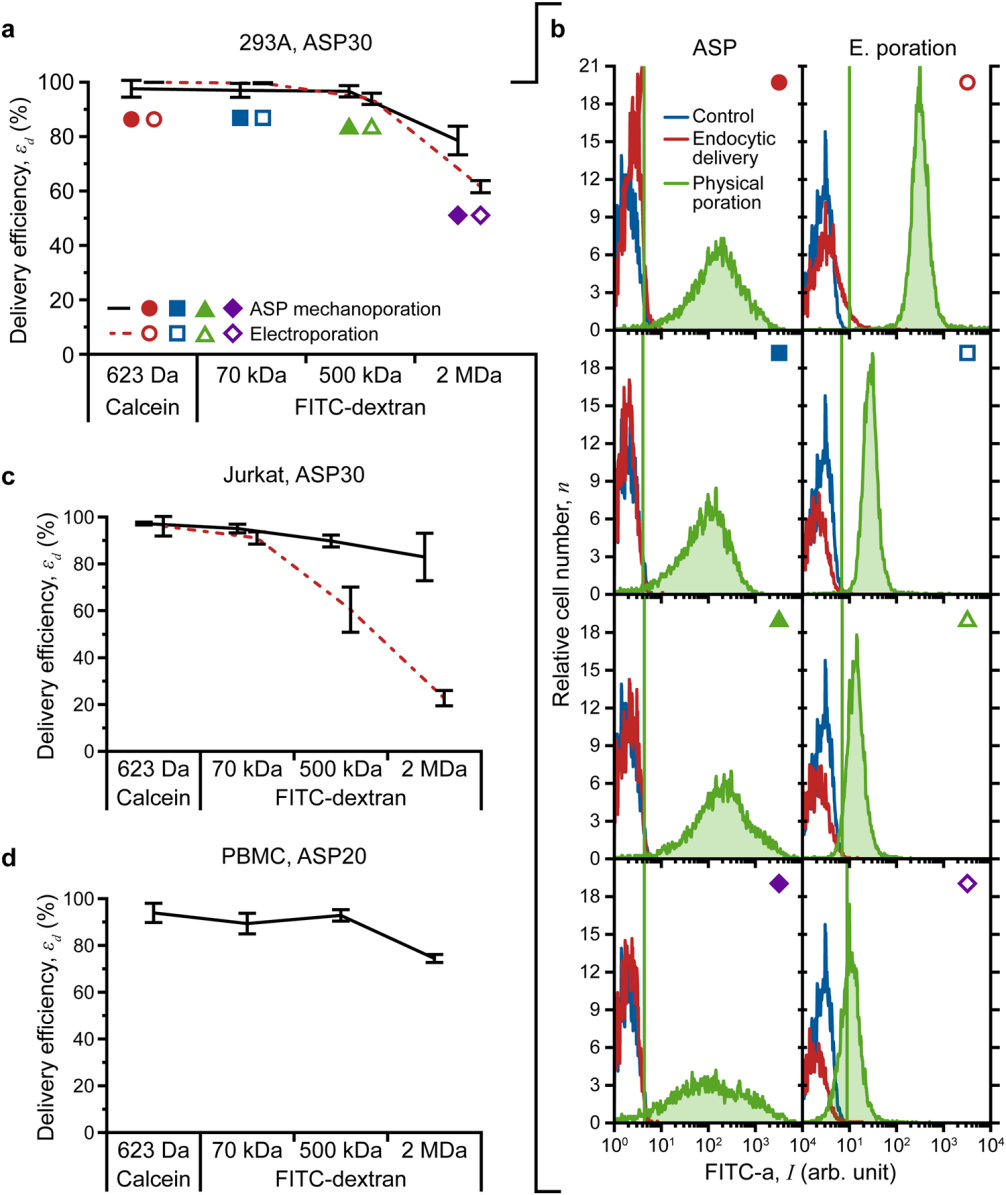
Quantitative assessment of macromolecule delivery performance: **(a)** comparison of ASP30 (30 μm orifice) and electroporation delivery efficiency for calcein, and 70 kDa, 500 kDa and 2 MDa FITC-labelled dextran into HEK 293 A cells, and **(b)** fluorescence intensity histograms (from flow cytometry) that illustrate method-specific differences in molecular uptake. **(c)** and **(d)** macromolecule delivery into Jurkat (ASP30 and electroporation) and peripheral blood mononuclear cells (PBMC, ASP20) isolated from whole-human blood, respectively. All data points were run in triplicate; error bars represent 2 SDs.

Flow cytometry results are summarized in Fig. 3. Target molecule uptake occurred following cell treatment by both methods (delivery efficiency defined as the fraction of live cells that take up the cargo; Supplementary Text). All ASP-treated cells (including PBMC) exhibited appreciable fluorescence across the range of molecules tested. In contrast, the electroporation method suffers a noticeable drop-off in uptake as the molecule size increases, particularly in Jurkat cells (Fig. 3c). Although the location of the fluorescence peak for ASP-treated 293 A cells remains constant for all conditions tested, peak broadening for the largest cargo molecules gives rise to a decrease in delivery efficiency from 96.6% (500 kDa FITC-dextran) to 78.6% (2 MDa). The fluorescence peak for electroporated cells remains narrow in all cases; however, it clearly shifts leftward with increasing molecular weight, with a corresponding decline in efficiency from 93.9% (500 kDa FITC-dextran) to 61.6% (2 MDa). Microscopy corroborates these observed trends (Supplementary Text and Supplementary Fig. S3). 293 A and Jurkat cells were treated with an ASP30 device (30 μm orifice); PBMC were treated with ASP20 (20 μm orifice). Both ASP and electroporation elicit a minimal decrease in viability, which remained >92% for all experimental conditions.

Flow cytometry and microscopy results reflect differences in the pore formation and resealing processes of the two methods. Electropermeabilization and mechanoporation occur on the time scale of microseconds; however, only small (<1 nm) pores persist beyond application of the electric field limiting the effectiveness of electroporation for large molecule delivery^2,49^. ASP treatment outcomes are consistent with mechanical permeabilization/diffusion-based delivery by sonoporation, which produces larger pores (20–500 nm) and exhibits prolonged membrane recovery times (seconds to minutes)^2,51,52^. Effective delivery of 2 MDa molecules implies an abundance of membrane pores as large as 54 nm in diameter^53^. Environmental scanning electron microscopy (ESEM) of ASP-treated 293 A cells supports this finding and indicates the persistence of some >100-nm diameter pores (Fig. 2b). Thus, indirect and direct measurements confirm formation of pores that are significantly larger (>50–100 nm) than typical electropores (1–25 nm)^49,54^.

As is common to most cytosolic delivery methods, ASP does not rely on exogenous materials, chemical alteration of the target molecule or an endocytic entry pathway. In addition, shear-based methods are not thought to damage sensitive cargo, which suggests that ASP is better suited to protein (and perhaps plasmid) delivery than electroporation^34^.

### Gene Transfection by Active Insertion

Membrane electroporation facilitates small molecule uptake by diffusion; however, gene transfer is likely aided by an electrophoretic effect that drives negatively charged DNA molecules across the membrane of perforated cells^54,55^. Typical exponentially decaying waveforms used for electroporation can be decomposed into membrane disruption (high-amplitude, short-duration (μs) pulse) and insertion (low-amplitude, long-duration (ms) tail) segments. ASP-mediated injury/diffusion-based delivery is demonstrated more effective than electroporation for macromolecules from 70 kDA (hydrodynamic radius *R*_*h*_ = ~6 nm) to 2 MDa (*R*_*h*_ = ~27 nm). While this result is evidence of a larger median pore size, improved delivery efficiency does not translate to transfection of plasmid DNA, which can easily exceed *R*_*h*_ = 50 nm. Accepting that electrophoretic migration of DNA to and into cells enhances gene transfer by electroporation, we hypothesized that exposing a mixture of mechanically-porated cells and plasmids to a low-amplitude, long duration electric field will improve transfection outcomes following ASP treatment.

Possessing a continuous flow configuration, ASP is adaptable for integration with pre-treatment, post-treatment and analytical steps in automated cell handling systems. This technology attribute also allows performance evaluation of a combined Acoustic Shear Poration-electrophoresis (ASP-EP) gene transfer method; the ASP mechanoporation system shown in Fig. 1 was coupled to a standard 2-mm gap electroporation cuvette. ASP-porated cells (HEK 293 A and Jurkat) were exposed to DNA plasmid expressing green fluorescent protein (pmaxGFP, 3.486 kbp, ~2.3 MDa, *R*_*h*_ > 50 nm^56^, Lonza) at two concentrations (30 and 50 μg/mL) with and without application of an electric field. Field strength was low enough to drive electrophoretic movement of DNA without additional cell membrane deformation. Because established cell lines are more readily transfected than primary cells, use of these lines facilitates detection of changes in transfection efficiency, however small. Further, as a shear-based physical method, ASP treatment should be cell-type agnostic so that results obtained with baseline cells translate to other cell types.

**Figure 4:**
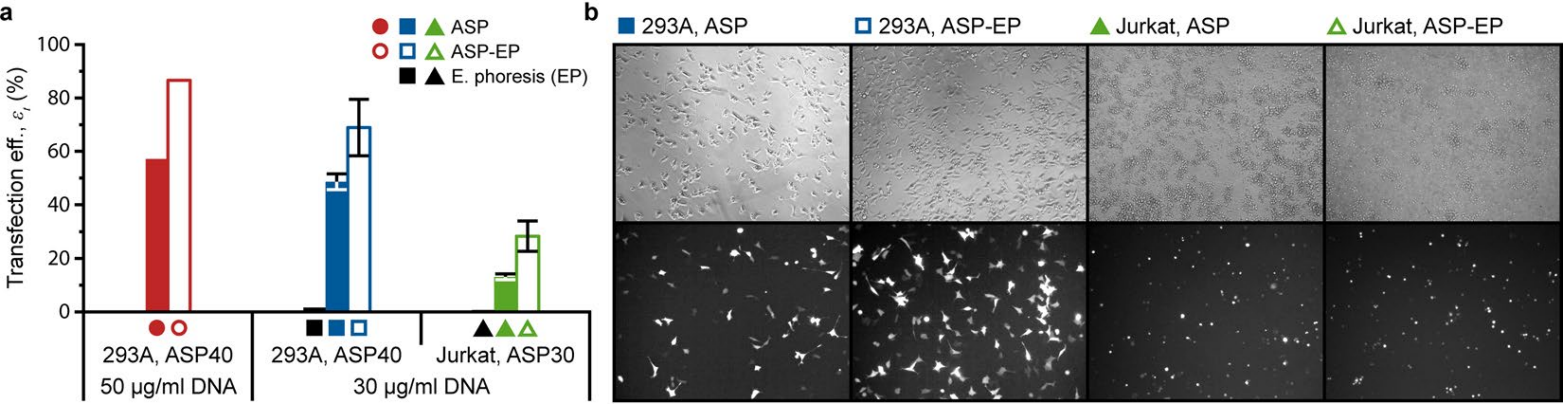
Assessment of Acoustic Shear Poration-electrophoresis (ASP-EP) transfection performance: **(a)** quantitative comparison of pmaxGFP transfection into HEK 293 A and Jurkat cells both with (ASP-EP) and without (ASP) application of a low-amplitude, long duration electrophoretic field following ASP mechanoporation, and **(b)** fluorescence microscopy indicates increased expression of green fluorescent protein (GFP) following combined-mode ASP-EP treatment. Data points with error bars were run in triplicate; error bars represent 2 SDs.

Figure 4 includes summary flow cytometry and fluorescence microscopy data for transfection of pmaxGFP plasmid into 293 A and Jurkat cells. Injury/diffusion-based delivery (ASP, solid bars/symbols) is less effective for the larger plasmid (*R*_*h*_ > 50 nm) than other macromolecules (*R*_*h*_ < ~27 nm) for both cell types. As expected of a purely diffusive entry mechanism, increasing the plasmid concentration augments ASP-mediated delivery/transfection of 293 A somewhat (from 49% to 57%). Performance of the multifunctional ASP-EP approach (open bars/symbols) is more remarkable. Addition of electrophoretic active insertion improved transfection efficiency for 293 A by 42–52%, and Jurkat transfection efficiency roughly doubled. Fluorescence microscopy provides visual confirmation of the flow cytometry results (Fig. 4b). 293 A were treated with an ASP40 device (40 μm orifice); Jurkat were treated with ASP30. Electrophoresis was performed in a commercial electroporation system (Multiporator, Eppendorf). Viability exceeded 87% for all experimental conditions suggesting that ASP-EP is no more stressful than ASP treatment alone. Coupling of ASP mechanoporation to an electrophoresis module increases the flexibility of the method and expands the range of potential macromolecule targets. ASP-EP overcomes pore size limitations of electropermeabilization while exploiting an electrophoretic effect to augment cytosolic delivery by diffusion.

## Discussion

We provide in-depth performance characterization of a hybrid intracellular delivery method that achieves sustained, transient membrane poration through short duration, high shear mechanical deformation of cells exposed to focused acoustic waves that force them through cell-scale orifices of a nozzle microarray^33,40–42^. Acoustic Shear Poration (ASP) enables efficient cytosolic delivery of large macromolecules (up to 2 MDa FITC-dextran, *R*_*h*_ = ~27 nm) into a variety of cell types, including difficult-to-transfect peripheral blood mononuclear cells. Based on molecular size alone, this result establishes the method’s capability for introduction of large molecular constructs including most proteins, antibodies and antigens, as well as small molecules and sensitive nanomaterials^57^. We also report for the first time combined permeabilization/active insertion-based transfection by exposing mechanoporated cells to an electric field that electrophoretically transports charged DNA to and into the cell interior. The ASP-EP approach not only substantially improves transfection outcomes, but it demonstrates the suitability of the ASP platform for larger scale process integration by coupling ASP and another treatment modality. ASP possesses great application flexibility with respect to target delivery cargo, and addition of EP provides another treatment dimension to enhance delivery of charged species. Finally, ultrasonic actuation and acoustic wave focusing enable access to low and high shear treatment domains relative to the state of the art, which expands the capability to work with cell types that are not well-served by existing methods. These attributes have the potential to impact research and discovery in a range of emerging clinical and laboratory applications. Of importance for industrial applications, the device and treatment method are capable of operation in a high-throughput, continuous flow format for treatment of large numbers of cells in a precisely controlled microfluidic environment.

## Methods

### System Fabrication and Experimental Setup

The silicon microarray fabrication process flow is detailed in our previous work^33,40,42^. Briefly, photolithographic patterning of plasma enhanced chemical vapor deposited (PECVD) silicon nitride dictated the size and arrangement of pyramidal nozzles anisotropically wet etched into silicon using a 45% w/w potassium hydroxide (KOH) solution. Acoustic Shear Poration (ASP) cartridge manufacture and assembly are described in Supplementary Text.

### Delivery Materials

Cell membrane-impermeant calcein (MW = 622.54 Da, hydrodynamic radius *R*_*h*_ = 0.74 nm; Molecular Probes) and assorted fluorescein isothiocyanate (FITC)-labelled dextran molecules were used as tracers of passive diffusion-based delivery. A 1 mM stock solution of calcein in phosphate-buffered saline without Ca^+2^ and Mg^+2^ ions (PBS) was diluted to a final concentration of 20 μM for use in uptake experiments. 70 kDa and 500 kDA (*R*_*h*_ = 6.5 nm and 15.9 nm, respectively; Sigma-Aldrich), and 2 MDa (*R*_*h*_ = 26.9 nm; Invitrogen) FITC-dextran molecules were suspended in PBS at 10 mg/mL. The stock concentration was adjusted to 0.5 mg/mL in the delivery buffer. Stock solutions were stored in a dark environment at −20 °C for up to one month. For transfection studies, pmaxGFP plasmid (*R*_*h*_ > 50 nm; Lonza), which encodes a green fluorescent protein (maxGFP), was added to cells at a final concentration of 30 μg/mL or 50 μg/mL depending on the experiment.

### Cell Culture, Primary Cell Isolation and Sample Preparation

Adherent cell line HEK 293 A cells (Invitrogen) were cultured in Dulbecco’s modification of Eagle’s medium (DMEM) (Cellgro) containing 4.5 g/L glucose and supplemented with 10% fetal bovine serum (FBS) (Cellgro). Suspension cell line human T lymphocyte Jurkat cells (ATCC) were cultured in Roswell Park Memorial Institute (RPMI 1640) medium (Cellgro) supplemented with 10% FBS. Peripheral blood mononuclear cells (PBMCs) were isolated using a previously described protocol^58^. All protocols were reviewed and approved by the Georgia Institute of Technology Institutional Review Board. All research was performed in accordance with relevant guidelines and regulations, and informed consent was received from all participants or their legal guardians. Freshly isolated PBMCs were suspended in RPMI 1640 with 10% FBS. Prior to testing, 293 A cells were detached from the surface of culture flasks using Trypsin EDTA 1 × 0.25% Trypsin/2.21 mM EDTA in HBSS without sodium bicarbonate, calcium and magnesium (Corning). For ASP-mediated delivery and transfection, cells (293 A, Jurkat and PBMC) were suspended in their respective complete growth media at a density of 2 × 10^6^ cells/mL in a volume of 800 μL. For electroporation experiments, cells were suspended in hypoosmolar electroporation buffer (Eppendorf) at recommended concentrations for each cell type (293 A, 2.5 × 10^6^ cells/mL; Jurkat, 1 × 10^6^ cells/mL). Fluorescent target molecules were added to the cell suspension at final concentrations immediately prior to device treatment.

### Cell Mechanoporation

Cell mechanoporation with diffusive uptake was conducted in triplicate for each fluorescent molecule (calcein and FITC-labelled dextran molecules of different molecular sizes). 293 A and Jurkat cells were treated with an ASP30 device (30 μm orifice); PBMC were treated with ASP20 (20 μm orifice). All mechanoporation experiments were run at the second acoustic resonance identified using a modeled harmonic response (see Supplementary Text and Supplementary Fig. S1, *f* = ~1.24–1.29 MHz). Cells were collected in a 1.5 mL microcentrifuge tube and allowed to rest for 15 min. Cell suspensions were then transferred to culture dishes containing growth media pre-equilibrated to 37 °C and held for 2 hrs before delivery assessment.

### Gene Transfection by Active Insertion

Transfection of pmaxGFP into 293 A and Jurkat cells was assessed following two treatment modalities, ASP cell mechanoporation as described above or combined Acoustic Shear Poration-electrophoresis (ASP-EP) whereby charged molecules were driven into mechanically porated cells under the action of a low amplitude electric field. In ASP-EP, cells were ejected directly into a 2-mm gap width electroporation cuvette, which was immediately placed in an Eppendorf Multiporator. A single 30 V, 500 μs pulse was applied to 293 A cells porated using an ASP40 device. The same signal waveform was used to treat Jurkat cells porated using an ASP30 device. Following ASP or ASP-EP treatment, cells rested for 10 minutes at room temperature. Cells were then added to 2 mL of culture media pre-equilibrated to 37 °C in the wells of a 6-well plate.

### Cell Electroporation

Manufacturer-recommended protocols were used to electropermeabilize cells for diffusive uptake of fluorescent molecules (Multiporator, Eppendorf). Again, experiments were conducted in triplicate. An 800 μL suspension of 293 A cells and target molecules at final concentrations was added to a 4-mm gap width electroporation cuvette, and a single 300 V exponentially decaying pulse with 50-μs time constant was applied. A 400 μL suspension of Jurkat cells and target molecules at final concentrations was added to a 2-mm gap width electroporation cuvette, and a single 240 V exponentially decaying pulse with 40-μs time constant was applied. After pulsing, cells were allowed to stand in the cuvette for 10 minutes at room temperature. Cells were transferred to culture dishes containing growth media pre-equilibrated to 37 °C and held for 2 hrs prior to analysis.

### Imaging and Flow Cytometry Analysis

Pore size was characterized directly using environmental scanning electron microscopy (ESEM) and indirectly by measuring delivery efficiency of various target molecules using fluorescence microscopy and flow cytometry. For ESEM measurements, ASP-treated samples were collected directly onto a glass slide. Mechanoporated cells were immediately transferred to an ESEM stub for evaluation of cell morphology and pore size measurements. Images were collected under a hydrated (humid) atmosphere (room temperature, 5 torr, 25 kV). Following treatment and the rest period, cells were washed two times with PBS to minimize background fluorescence before plating at 0.3 mL per well of a 24-well plate for microscopy and flow cytometry. Delivery efficiency and loss of cell viability were quantified by flow cytometry (BD LSR II, Becton Dickinson) using BD FACSDiva software. Viable cells were distinguished from non-viable cells by adding 5 μL of 7-Amino-actinomycin D (7-AAD, BioLegend) to cell suspensions. 7-AAD is excluded by viable cells but penetrates cell membranes of dying or dead cells and undergoes a spectral shift after association with DNA. A 488 nm argon laser was used for excitation. Uptake and viability were detected using appropriate filters (FITC, green, 530/30 nm; 7-AAD, red, 695/40 nm). At least 10,000 viable cells were analyzed per sample, and collected data was processed and analyzed using Flowing 2^59^. Untreated, target-molecule free samples served as controls for background fluorescence and cell viability. Untreated cells incubated for 10 min with the respective fluorescent molecules were used as an additional control for detecting non-specific binding. Post-transfection analyses were carried out after 24 hr. Cells transfected with pmaxGFP were visually inspected for expression of GFP using fluorescence microscopy; cell viability and transfection efficiency were quantified using flow cytometry as described above for passive diffusive uptake studies.

### Data Availability

The datasets generated and/or analyzed during the current study are available from the corresponding author on reasonable request.

## Electronic supplementary material

Supplementary Information

## References

[1] Singer SJ, Nicolson GL (1972). The fluid mosaic model of the structure of cell membranes. Science.

[2] Meacham JM, Durvasula K, Degertekin FL, Fedorov AG (2014). Physical methods for intracellular delivery: Practical aspects from laboratory use to industrial-scale processing. Journal of laboratory automation.

[3] Stewart MP (2016). *In vitro* and *ex vivo* strategies for intracellular delivery. Nature.

[4] Kim D (2009). Generation of human induced pluripotent stem cells by direct delivery of reprogramming proteins. Cell stem cell.

[5] Ramakrishna S, Kim KS, Baek KH (2014). Posttranslational modifications of defined embryonic reprogramming transcription factors. Cellular reprogramming.

[6] Thier M, Munst B, Mielke S, Edenhofer F (2012). Cellular reprogramming employing recombinant Sox2 protein. Stem cells international.

[7] Kim S, Kim D, Cho SW, Kim J, Kim JS (2014). Highly efficient RNA-guided genome editing in human cells via delivery of purified Cas9 ribonucleoproteins. Genome research.

[8] Michalet X (2005). Quantum dots for live cells, *in vivo* imaging, and diagnostics. Science.

[9] Lukacs GL (2000). Size-dependent DNA mobility in cytoplasm and nucleus. Journal of biological chemistry.

[10] Belting M, Sandgren S, Wittrup A (2005). Nuclear delivery of macromolecules: Barriers and carriers. Advanced drug delivery reviews.

[11] Lechardeur D, Verkman AS, Lukacs GL (2005). Intracellular routing of plasmid DNA during non-viral gene transfer. Advanced drug delivery reviews.

[12] Vaughan EE, DeGiulio JV, Dean DA (2006). Intracellular trafficking of plasmids for gene therapy: Mechanisms of cytoplasmic movement and nuclear import. Current gene therapy.

[13] Kay MA, Glorioso JC, Naldini L (2001). Viral vectors for gene therapy: the art of turning infectious agents into vehicles of therapeutics. Nature medicine.

[14] Waehler R, Russell SJ, Curiel DT (2007). Engineering targeted viral vectors for gene therapy. Nature reviews genetics.

[15] Hacein-Bey-Abina S (2003). A serious adverse event after successful gene therapy for X-linked severe combined immunodeficiency. New England journal of medicine.

[16] Hacein-Bey-Abina S (2003). LMO2-associated clonal T cell proliferation in two patients after gene therapy for SCID-X1. Science.

[17] Elouahabi A, Ruysschaert JM (2005). Formation and intracellular trafficking of lipoplexes and polyplexes. Molecular therapy.

[18] Torchilin VP (2006). Recent approaches to intracellular delivery of drugs and DNA and organelle targeting. Annual review of biomedical engineering.

[19] Heitz F, Morris MC, Divita G (2009). Twenty years of cell-penetrating peptides: from molecular mechanisms to therapeutics. British journal of pharmacology.

[20] Hyodo M, Sakurai Y, Akita H, Harashima H (2014). “Programmed packaging” for gene delivery. Journal of controlled release.

[21] Capecchi MR (1980). High-efficiency Transformation by Direct Micro-injection of DNA into Cultured Mammalian Cells. Cell.

[22] Zhang Y, Yu LC (2008). Microinjection as a tool of mechanical delivery. Current opinion in biotechnology.

[23] Neumann E, Schaeferridder M, Wang Y, Hofschneider PH (1982). Gene Transfer into Mouse Lyoma Cells by Electroporation in High Electric Fields. EMBO journal.

[24] Wolff JA (1990). Direct Gene Transfer into Mouse Muslce *In Vivo*. Science.

[25] Jantsch J (2008). Small interfering RNA (siRNA) delivery into murine bone marrow-derived dendritic cells by electroporation. Journal of immunological methods.

[26] Wiese M (2010). Small interfering RNA (siRNA) delivery into murine bone marrow-derived macrophages by electroporation. Journal of immunological methods.

[27] Lambert H, Pankov R, Gauthier J, Hancock R (1990). Electroporation-mediated Uptake of Proteins into Mammalian Cells. Biochemistry and cell biology-Biochimie et biologie cellulaire.

[28] Chakravarty P, Qian W, El-Sayed MA, Prausnitz MR (2010). Delivery of molecules into cells using carbon nanoparticles activated by femtosecond laser pulses. Nature nanotechnology.

[29] Wu TH (2011). Photothermal Nanoblade for Large Cargo Delivery into Mammalian Cells. Analytical chemistry.

[30] Yoon S (2016). Direct and sustained intracellular delivery of exogenous molecules using acoustic-transfection with high frequency ultrasound. Scientific reports.

[31] Zhang ZX (2017). Hypersonic Poration: A New Versatile Cell Poration Method to Enhance Cellular Uptake Using a Piezoelectric Nano-Electromechanical Device. Small.

[32] Hallow DM (2008). Shear-induced intracellular loading of cells with molecules by controlled microfluidics. Biotechnology and bioengineering.

[33] Zarnitsyn VG (2008). Electrosonic ejector microarray for drug and gene delivery. Biomedical microdevices.

[34] Sharei A (2013). A vector-free microfluidic platform for intracellular delivery. Proceedings of the National Academy of Sciences of the United States of America.

[35] Szeto GL (2015). Microfluidic squeezing for intracellular antigen loading in polyclonal B-cells as cellular vaccines. Scientific reports.

[36] Sharei A (2015). *Ex Vivo* Cytosolic Delivery of Functional Macromolecules to Immune Cells. PLOS One.

[37] Geyer MB, Brentjens RJ (2016). Review: Current clinical applications of chimeric antigen receptor (CAR) modified T cells. Cytotherapy.

[38] Meacham JM, Durvasula K, Fedorov AG, Degertekin FL, Mehta A (2017). Intracellular delivery and transfection methods and devices. USPTO pat..

[39] Ding, X. *et al*. High-throughput nuclear delivery and rapid expression of DNA via mechanical and electrical cell-membrane disruption. *Nature biomedical engineering* **1**, 0039, 10.1038/s41551-017-0039,https://www.nature.com/articles/s41551-017-0039#supplementary-information (2017). 10.1038/s41551-017-0039 PMC5602535 28932622

[40] Meacham JM, Ejimofor C, Kumar S, Degertekin FL, Fedorov AG (2004). Micromachined ultrasonic droplet generator based on a liquid horn structure. Review of scientific instruments.

[41] Meacham JM, Varady MJ, Esposito D, Degertekin FL, Fedorov AG (2008). Micromachined ultrasonic atomizer for liquid fuels. Atomization and sprays.

[42] Meacham JM, Varady MJ, Degertekin FL, Fedorov AG (2005). Droplet formation and ejection from a micromachined ultrasonic droplet generator: Visualization and scaling. Physics of fluids.

[43] Lokhandwalla M, Sturtevant B (2001). Mechanical haemolysis in shock wave lithotripsy (SWL): I. Analysis of cell deformation due to SWL flow-fields. Physics in medicine and biology.

[44] Wolfe J, Dowgert MF, Steponkus PL (1986). Mechanical Study of the Deformation and Rupture of the Plasma Membranes of Protoplasts During Osmotic Expansions. Journal of membrane biology.

[45] Needham D, Nunn RS (1990). Elastic Deformation and Failure of Lipid Bilayer Membranes Containing Cholesterol. Biophysical journal.

[46] Olbrich K, Rawicz W, Needham D, Evans E (2000). Water permeability and mechanical strength of polyunsaturated lipid bilayers. Biophysical journal.

[47] Evans E, Heinrich V, Ludwig F, Rawicz W (2003). Dynamic tension spectroscopy and strength of biomembranes. Biophysical journal.

[48] Gehl J (2003). Electroporation: theory and methods, perspectives for drug delivery, gene therapy and research. Acta physiologica Scandinavica.

[49] Krassowska W, Filev PD (2007). Modeling electroporation in a single cell. Biophysical journal.

[50] Gabriel B, Teissie J (1997). Direct observation in the millisecond time range of fluorescent molecule asymmetrical interaction with the electropermeabilized cell membrane. Biophysical journal.

[51] Karshafian R, Samac S, Bevan PD, Burns PN (2010). Microbubble mediated sonoporation of cells in suspension: Clonogenic viability and influence of molecular size on uptake. Ultrasonics.

[52] Duvshani-Eshet M, Baruch L, Kesselman E, Shimoni E, Machluf M (2006). Therapeutic ultrasound-mediated DNA to cell and nucleus: bioeffects revealed by confocal and atomic force microscopy. Gene therapy.

[53] Armstrong JK, Wenby RB, Meiselman HJ, Fisher TC (2004). The hydrodynamic radii of macromolecules and their effect on red blood cell aggregation. Biophysical journal.

[54] Sukharev SI, Klenchin VA, Serov SM, Chernomordik LV, Chizmadzhev YA (1992). Electroporation and Electrophoretic DNA Transfer into Cells–The Effect of DNA Interaction with Electropores. Biophysical journal.

[55] Dimitrov DS, Sowers AE (1990). Membrane Electroporation–Fast Molecular Exchange by Electroosmosis. Biochimica et biophysica acta.

[56] Fishman DM, Patterson GD (1996). Light scattering studies of supercoiled and nicked DNA. Biopolymers.

[57] Hartmann WK, Saptharishi N, Yang XY, Mitra G, Soman G (2004). Characterization and analysis of thermal denaturation of antibodies by size exclusion high-performance liquid chromatography with quadruple detection. Analytical biochemistry.

[58] Keegan PM, Surapaneni S, Platt MO (2012). Sickle Cell Disease Activates Peripheral Blood Mononuclear Cells to Induce Cathepsins K and V Activity in Endothelial Cells. Anemia.

[59] Terho, P. Flowing 2. Centre for Biotechnology, University of Turku, Finlind (2016).

