# Supplementary material for: Enhanced intracellular delivery via coordinated acoustically driven shear mechanoporation and electrophoretic insertion
Source: Sci Rep. 2018 Feb 27;8:3727. doi: 10.1038/s41598-018-22042-0 (PMC5829135; doi:10.1038/s41598-018-22042-0)
Supplement: Supplementary file 1 — Supplementary Information [file 41598_2018_22042_MOESM1_ESM.pdf]

Enhanced intracellular delivery via coordinated acoustically driven shear mechanoporation and electrophoretic insertion

J. Mark Meacham[1], Kiran Durvasula[2], F. Levent Degertekin[3,4,5], and Andrei G. Fedorov[4,5]

[1]Department of Mechanical Engineering and Materials Science, Washington University in St. Louis, St. Louis, MO 63130; [2]OpenCell Technologies, Inc., St. Louis, MO 63108; [3]School of Electrical and Computer Engineering, [4]Parker H. Petit Institute for Bioengineering and Bioscience, and [5]G. W. Woodruff School of Mechanical Engineering, Georgia Institute of Technology, Atlanta, GA 30332. Correspondence and requests for materials should be addressed to J.M.M. (email: meachamjm@wustl.edu)

This file includes: Supplementary Text and Supplementary Figures S1 to S4.

Supplementary Text

Acoustic Shear Poration (ASP) Harmonic Response Analysis. Finite element analysis (FEA) was used to predict the harmonic response of the assembled ASP device[1,2]. Supplementary Figure S1 illustrates the computational domain, a two-dimensional (2D) cross section of assembled components with subdomains for the piezoelectric transducer (ANSYS® Mechanical APDL PLANE223 coupled field piezoelectric elements[2]), polymer cartridge and nozzle microarray (PLANE183 structural solid), and fluid (FLUID29 harmonic acoustic fluid). Geometry is as shown in Supplementary Fig. 1, i.e., the dimensions of the various components are taken from the experimental setup. Piezoelectric thickness t_P = 1.5 mm and width w_P = 24 mm, chamber height h_c = 1.5 mm (from piezoelectric surface to nozzle orifice) and width w_c = 5 mm, and microarray thickness t_{Si} = 500 μm, which corresponds to the thickness of a standard silicon wafer (note: due to symmetry, the computational domain represents half of the device cross section; the given widths are for the complete assembly). Piezoelectric material properties (permittivity $\boldsymbol{\varepsilon}$, piezoelectric coupling matrix $\mathbf{e}$ and stiffness tensor $\mathbf{c_E}$) were obtained from the vendor (for P880, American Piezo Ceramics) and the literature[3]. Physical properties of silicon, polycarbonate and water (to represent cell media) are widely available.

The model piezoelectric was driven at a constant voltage V_P = 10 V_{pp} while sweeping the frequency f from 0.5 to 2.0 MHz. Resonant frequencies of operation were identified by analyzing relevant frequency-dependent operating characteristics (electrical input impedance and normalized tip pressure gradient). The impedance response (inset, Supplementary Fig. S1) predicted several device resonances (local minima/maxima); however, it was difficult to determine which frequencies corresponded to sample ejection (and cell treatment) due to the relatively minor influence of fluid sample loading on the device response. These frequencies were easily identified by plotting the average nozzle tip pressure gradient versus frequency of operation (Supplementary Fig. S1). FEA also allowed virtual observation of the spatial displacement (within solids) and acoustic pressure (within liquids) fields at any frequency of interest. For example, Supplementary Fig. S1 includes a contour plot of the amplitude of the pressure field within the ejection chamber at its second half-wave resonance (n = 2). The contour plot confirms the pressure distribution expected of operation at the second ejection mode, i.e., roughly linear striations with a full pressure wavelength maintained between the piezoelectric surface and nozzle focal point[1].

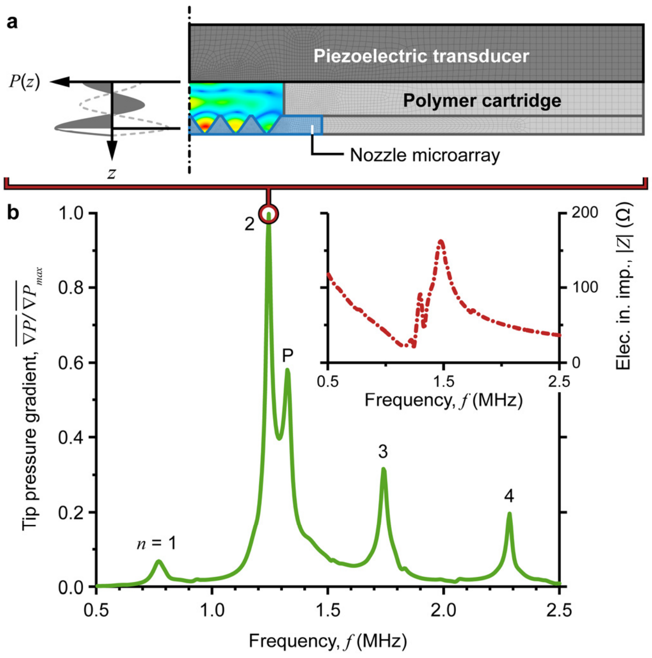

Supplementary Fig. S1, finite element analysis (FEA) of ASP harmonic response: **(a)** device half domain illustrating piezoelectric transducer, polymer cartridge, nozzle microarray and fluid ejection chamber subdomains. The predicted pressure field for a drive frequency of 1.25 MHz indicates operation at the device second half-wave resonance that corresponds to ejection. **(b)** average tip pressure gradient and electrical input impedance (inset) versus operating frequency. The first four half-wave resonances (n = 1, 2, 3 and 4) and the natural longitudinal resonance of the piezoelectric transducer (P) are noted.

Device Fabrication, Assembly and Operation. The external sample reservoir, channels and ejection chamber were cut into 125- and 250-μm thick polycarbonate sheets (McMaster Carr) using a CO_2 laser cutter/engraver (Speedy 300, Trotec). The piezoelectric transducer (P880, American Piezo Ceramics), silicon microarray and reservoir layers were assembled using acrylic adhesive transfer tape (McMaster Carr) such that the height of the ejection chamber corresponded to a desired resonant frequency of operation (aqueous solution, speed of sound c ~1500 m/s, desired frequency f ~1 MHz, chamber height $h_c \approx n\, c/(2\,f) \approx 1.5$ mm (for $n = 2$)). The microfluidic reservoir/chamber assembly was fastened to laser cut 6.35-mm thick acrylic structural components. A simple analytical expression provides a rough guideline for device design; however, exact operating frequencies were predicted using the FEA model and confirmed experimentally. For the device shown in Supplementary Fig. S2, three polycarbonate layers were used (total thickness of 900 μm including adhesive). Accounting for the 500 μm thick silicon microarray, a second half-wave resonance of ~1.25 MHz was found (based on observed sample ejection, Supplementary Fig. S1; FEA predicted resonance at f = 1.245 MHz). Sample loaded at the device inlet passes through the ejection chamber into the external reservoir. After loading a prescribed volume, piezoelectric actuation (33522A, Agilent; 2100L, Electronic Navigation Industries) draws fluid from this reservoir as it expels sample from the orifices of the microarray (Supplementary Fig. S2). Additional details of the microarray geometry are shown in the scanning electron micrographs (SEM) of Supplementary Fig. S2.

Shear-based Mechanoporation Treatment Domains. Hallow et al.[4] report delivery of various molecules (calcein, FITC-dextran and bovine serum albumin (BSA)) into human prostate cancer cells (DU145). Cells were exposed to controlled shear as cell mixtures were forced (by syringe

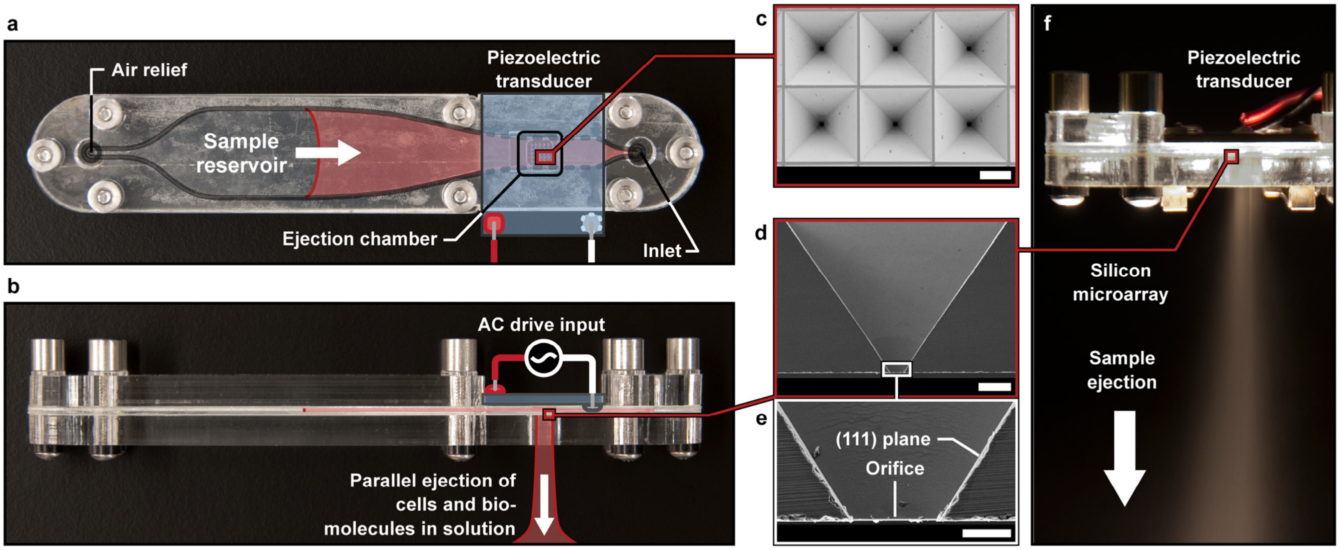

Supplementary Fig. S2, ASP cartridge assembly and operation: **(a,b)** plan- and side-view of assembled ASP module, which illustrates sample reservoir emptying and ejection from the nozzle microarray, **(c-e)** SEM images of a representative microarray with 42 µm orifices (scale bars are 300, 100 and 25 µm, respectively), and **(f)** sample misting from an array of 42 µm orifices at a drive frequency of 1.25 MHz.

pump or hand-actuated syringe) through a single cylindrical orifice ([37a]) or an array of conical orifices ([37b]) drilled into Mylar sheets. For the single-orifice configuration [37a], devices were fabricated with 50, 60, 70 and 80-µm diameter orifices. The length l of the cylindrical orifices was dictated by the thickness of the Mylar film, either 100 or 250 µm. Velocity was varied from ~1.7 to 14 m/s (30 to 100 mL/hr). For this device, time of treatment ($\tau \sim l/U$) scales with channel length (as opposed to orifice diameter) with minimum and maximum values of 7.0×10^{-6} s and 1.5×10^{-4} s, respectively. Shear rate ($k \sim U/(d/2)$) ranged from 4.1×10^{4} s^{-1} to 5.7×10^{5} s^{-1}. 3×3 arrays of tapered conical orifices (device configuration [37b]) were made with 40 and 50-µm diameter orifices. For the same flow rates used with the single orifice plate (30 to 100 mL/hr), velocity varied from 0.7 to 2.5 m/s. Time of treatment was 1.6×10^{-5} s to 1.0×10^{-4} s and maximum shear rate was 1.8×10^{4} s^{-1} to 1.2×10^{5} s^{-1}.

Sharei et al.[5] report delivery of numerous macromolecules and nanoparticles (cascade blue-labelled dextrans, decorated carbon nanotubes, antibodies, transcription factors) into laboratory established (HeLa), primary and stem cells. Mechanoporation of cells was achieved by controlled flow (pressure-regulated) through single or a series of sub cell-sized microchannel constrictions. Here, calculation of the treatment domain is based on treatment of Hela cells, for which the most complete dataset is available. Devices were fabricated with ranges of constriction width (4 and 8 µm) and length (10, 20 and 40 µm), and flow velocity was varied from 100 to 600 mm/s. Determination of the k-τ space for a particular method is an order of magnitude analysis; however, the device configuration of Sharei et al.[5] highlights the importance of selecting an appropriate length scale. In the microchannel constrictions, fluid is confined to a thin layer between the cell and wall, and so use of constriction half width would yield an unrealistic estimate of the shear rate. The shear layer thickness is unknown; therefore, we use 1/10 of the width in calculating attainable shear rates for this method. Under these operating conditions, time of treatment was 6.7×10^{-5} s to 4.0×10^{-4} s and shear rate was 2.5×10^{4} s^{-1} to 3.0×10^{5} s^{-1}.

Assessing Mechanoporation and Intracellular Delivery of Fluorescent Molecules by Passive Diffusion. Supplementary Figure S3 is a comparison of ASP- and electroporation-mediated uptake of fluorescent molecules (calcein, and 70 kDa, 500 kDa and 2 MDa FITC-labelled dextran) into HEK 293A cells. Robust fluorescence is observed for ASP-treated cells across all cargo molecules yielding only a slight decrease in fluorescence intensity with increasing molecule size. Similar results were obtained for Jurkat and peripheral blood mononuclear cells (PBMC). Efficient delivery is also seen with calcein and 70 kDa FITC-dextran in electroporated 293A cells; however, Supplementary Fig. S3 shows a marked decrease in uptake of higher molecular weight FITC-dextran molecules (500 kDa and 2 MDa) for this cell type following electropermeabilization. Microscopy suggests that electroporated cells do not take up any 2-MDa FITC-labelled dextran; however, flow cytometry indicates that the loss of fluorescence intensity is due to a dramatic reduction in the number of delivered molecules per cell and not complete exclusion of these molecules (Fig. 3).

Supplementary Figure S4 is a comparison of fluorescence intensity histograms of control/no target 293A cells, control/endocytosis 293A cells exposed to 2 MDa FITC-dextran for 10 min, and 293A treated using an ASP30 device. To account for non-specific binding and endocytosis, the lower bound on the delivery region (shaded green) was defined to include the top 0.5% of live control cells for FITC-dextrans; a 5% threshold was used for the small molecule calcein, which exhibits slight but measurable endocytic uptake and/or non-specific binding.

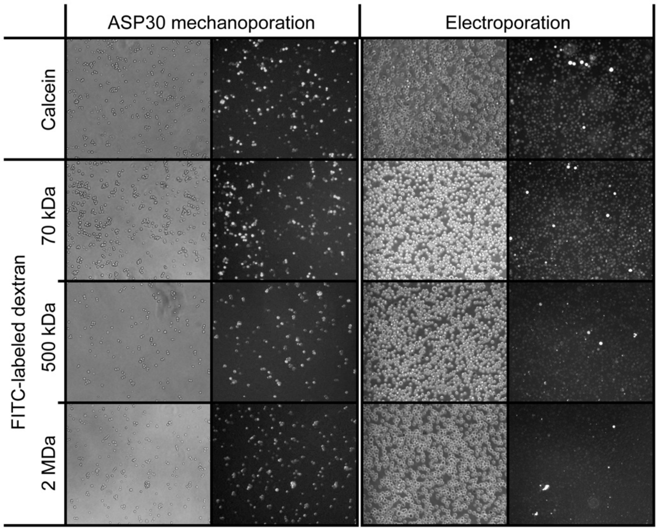

Supplementary Fig. S3, qualitative assessment of macromolecule delivery performance using fluorescence microscopy. Images demonstrate delivery of calcein, and 70 kDa, 500 kDa and 2 MDa FITC-labelled dextran into HEK 293A cells using ASP30 (30 µm orifice) and electroporation on the left and right, respectively. Images are corrected for brightness and contrast using ImageJ[6]. Microscopy is not used for quantitative assessment of molecular uptake.

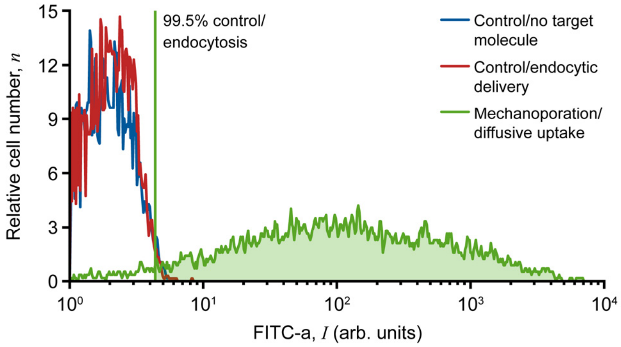

Supplementary Fig. S4, quantitative assessment of macromolecule delivery using flow cytometry: comparison of fluorescence intensity histograms for control/endocytic delivery and treated samples defines uptake (5% threshold of control for calcein; 0.5% threshold for FITC-dextran molecules). Data for delivery of 2 MDa FITC-dextran into HEK 293A cells using an ASP30 (30 µm orifice) are shown.

References

1 Meacham, J. M., Varady, M. J., Esposito, D., Degertekin, F. L. & Fedorov, A. G. Micromachined ultrasonic atomizer for liquid fuels. *Atomization and sprays* **18**, 163-190, doi:10.1615/AtomizSpr.v18.i2.30 (2008).

2 ANSYS, I. ANSYS Academic Research Mechanical APDL Release 15.0 (2014).

3 Kino, G. S. Acoustic Waves: Devices, Imaging, and Analog Signal Processing. (Prentice Hall, 1987).

4 Hallow, D. M. *et al.* Shear-induced intracellular loading of cells with molecules by controlled microfluidics. *Biotechnology and bioengineering* **99**, 846-854, doi:10.1002/bit.21651 (2008).

5 Sharei, A. *et al.* A vector-free microfluidic platform for intracellular delivery. *Proceedings of the National Academy of Sciences of the United States of America* **110**, 2082-2087, doi:10.1073/pnas.1218705110 (2013).

6 Rasband, W. S. ImageJ. U. S. National Institutes of Health, Bethesda, MA, USA (2016).
